# Supplementary material for: The aging mouse CNS is protected by an autophagy-dependent microglia population promoted by IL-34
Source: Nat Commun. 2024 Jan 9;15:383. doi: 10.1038/s41467-023-44556-6 (PMC10776874; doi:10.1038/s41467-023-44556-6)
Supplement: Supplementary file 1 — Supplementary Information [file 41467_2023_44556_MOESM1_ESM.docx]

Supplementary figures


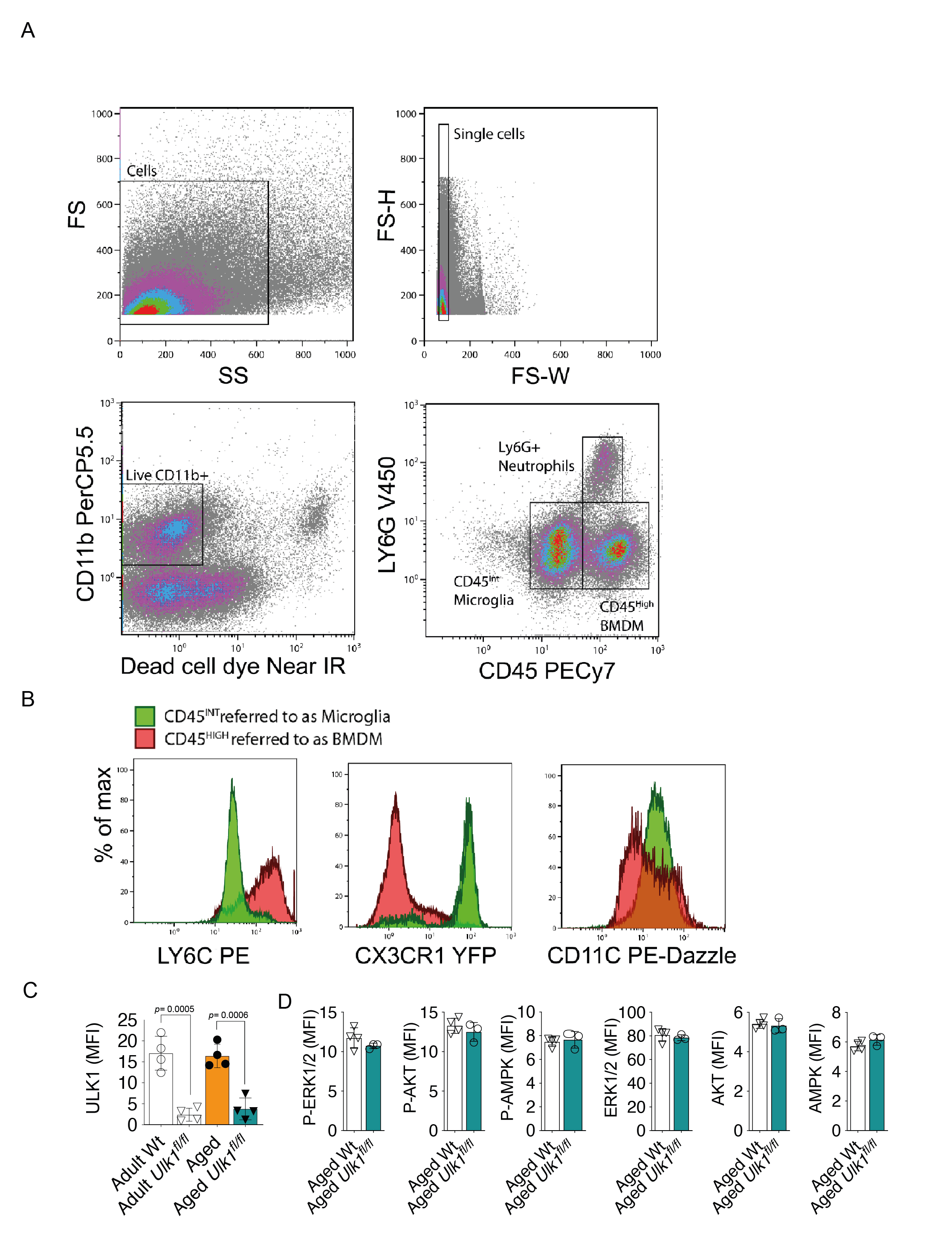


Supplementary Figure 1

(A) Myeloid and microglia flow cytometry gating strategy.

(B) Flow cytometry MFI histograms.

(C) ULK1 expression in microglia assessed by flow cytometry (all conditions *n*=4). Analyses separated for adult and aged.

(D) Phosphorylation state and total protein content in microglia (Aged Wt, *n*=4; Aged *Ulk1^fl/fl^*, *n*=3) after Tamoxifen-induced *Ulk1* deletion in vitro quantified by flow cytometry.

(C) – (D) Two-tailed unpaired T-test. Error bars represent mean + SD. Source data are provided as a Source Data file.


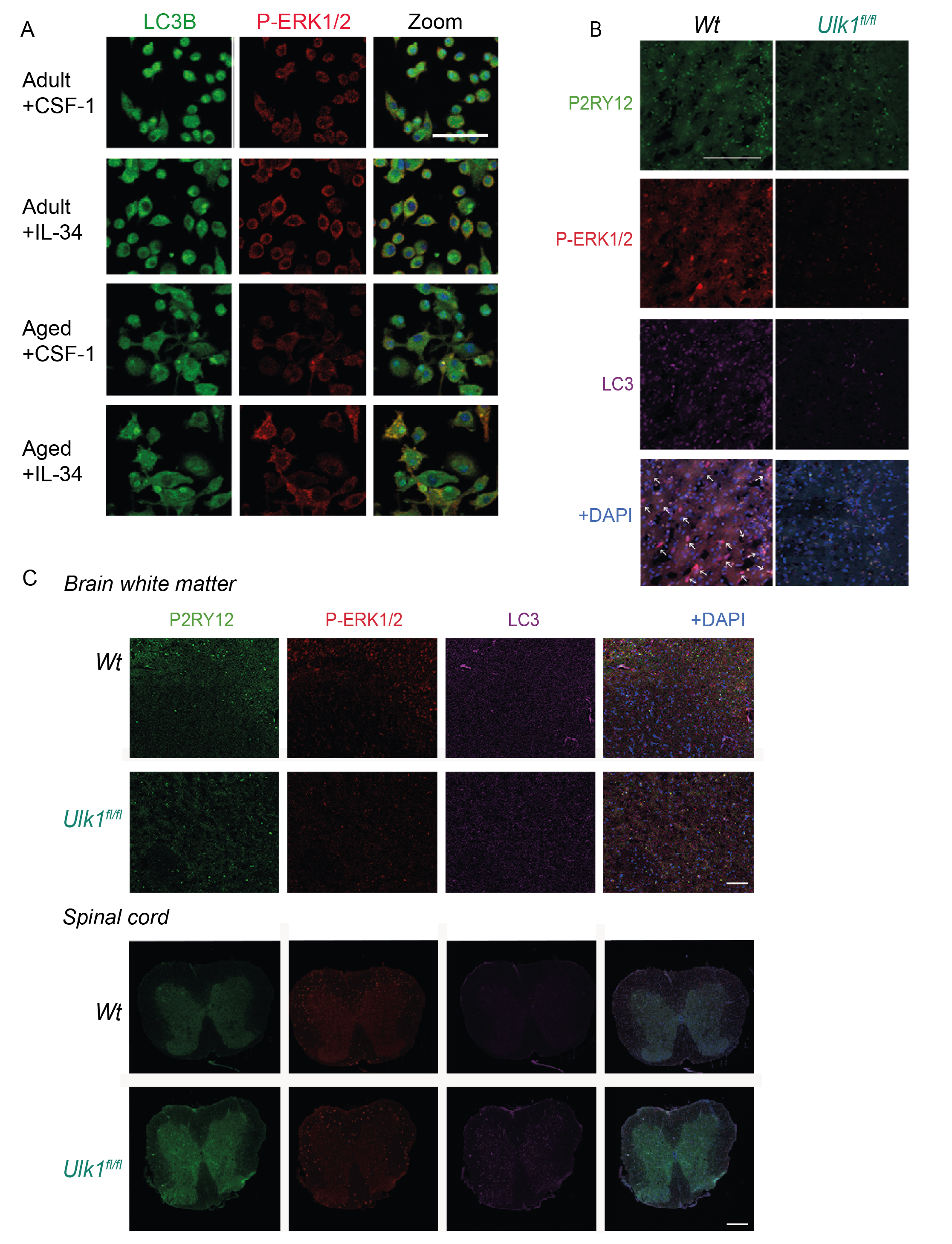


Supplementary figure 2

(A) Zoom of immunocytochemistry images from figure 3A. Scale bar represent 50 μm

(B) Immunohistochemistry images of microglia in brain cortical regions. Arrows indicate P-ERK1/2^High^ cells. Zoom of image from figure 4G. Scale bar represent 100 μm

(C) Representative immunohistochemistry images of microglia in white matter and spinal cord regions. Quantification shown in figure 4H. Scale bars represent 100 μm in images of *Brain white matter* and 200 μm in images of *Spinal cord.*


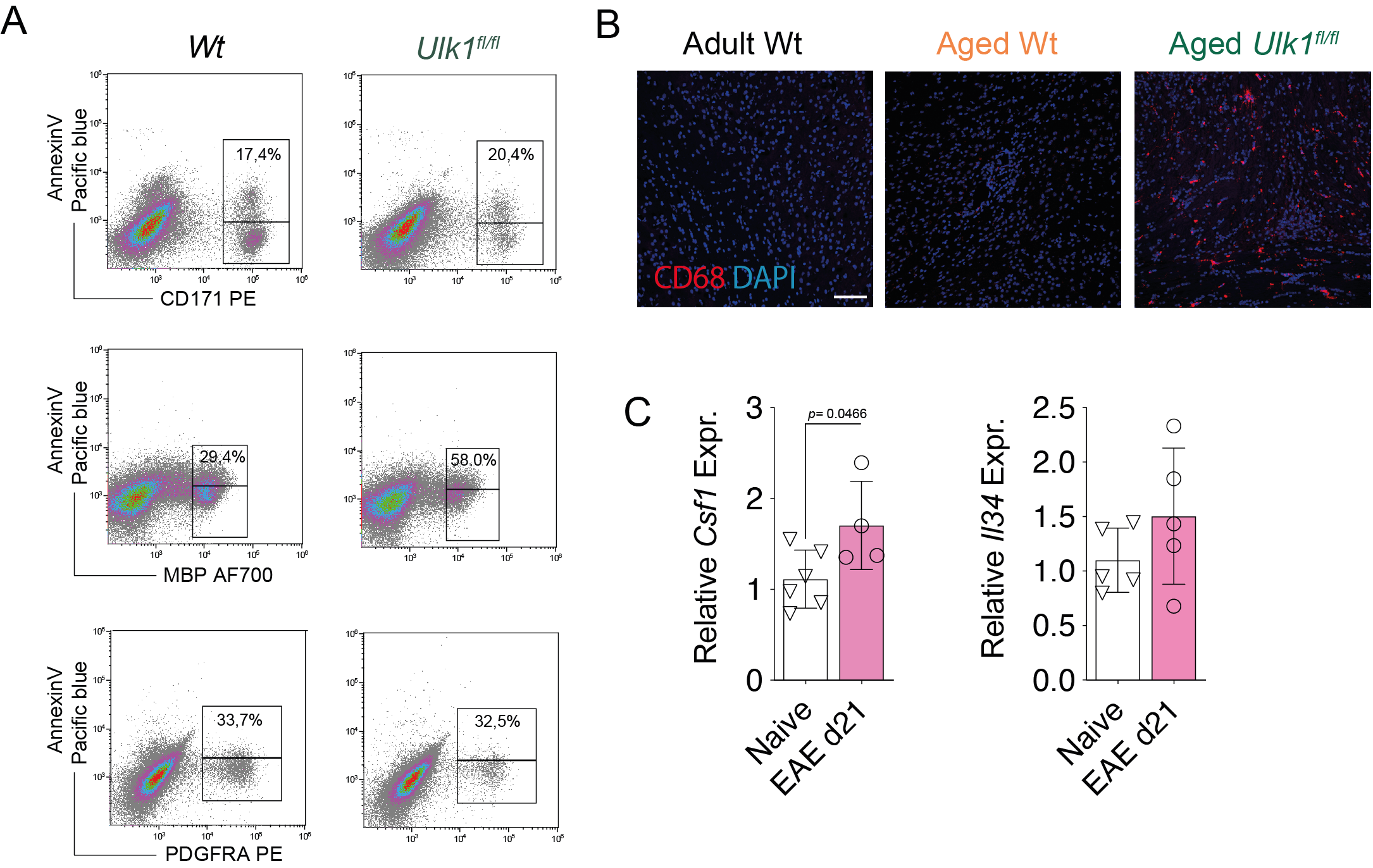


Supplementary figure 3

(A) Representative flow cytometry plots for figure 6B analysis.

(B) Representative immunohistochemical images of brain parenchyma outside lesions day 15 post EAE induction (quantification shown in figure 6C). Scale bars correspond to 250 μm.

(C) mRNA expression of *Il34* and *Csf*1 in CNS during EAE 21 days after immunization (*Csf1,* Wt aged, *n*=4; *Ulk1^fl/fl^* , *n*=5 and *Il34*, Wt aged, *n*=5; *Ulk1^fl/fl^* , *n*=5).

Adult mice were 3-5-month-old, < 1 month post-Tamoxifen treatment, Aged mice were > 20 months old, > 18 months post Tamoxifen treatment. *Ulk1^fl/fl^* refer to *Ulk1^fl/fl^* CX3CR1^CreERT2^ *and Wt* to *Ulk1^wt/wt^* CX3CR1^CreERT2^. (C) Two-tailed unpaired T-test. Error bars represent mean + SD. Source data are provided as a Source Data file.


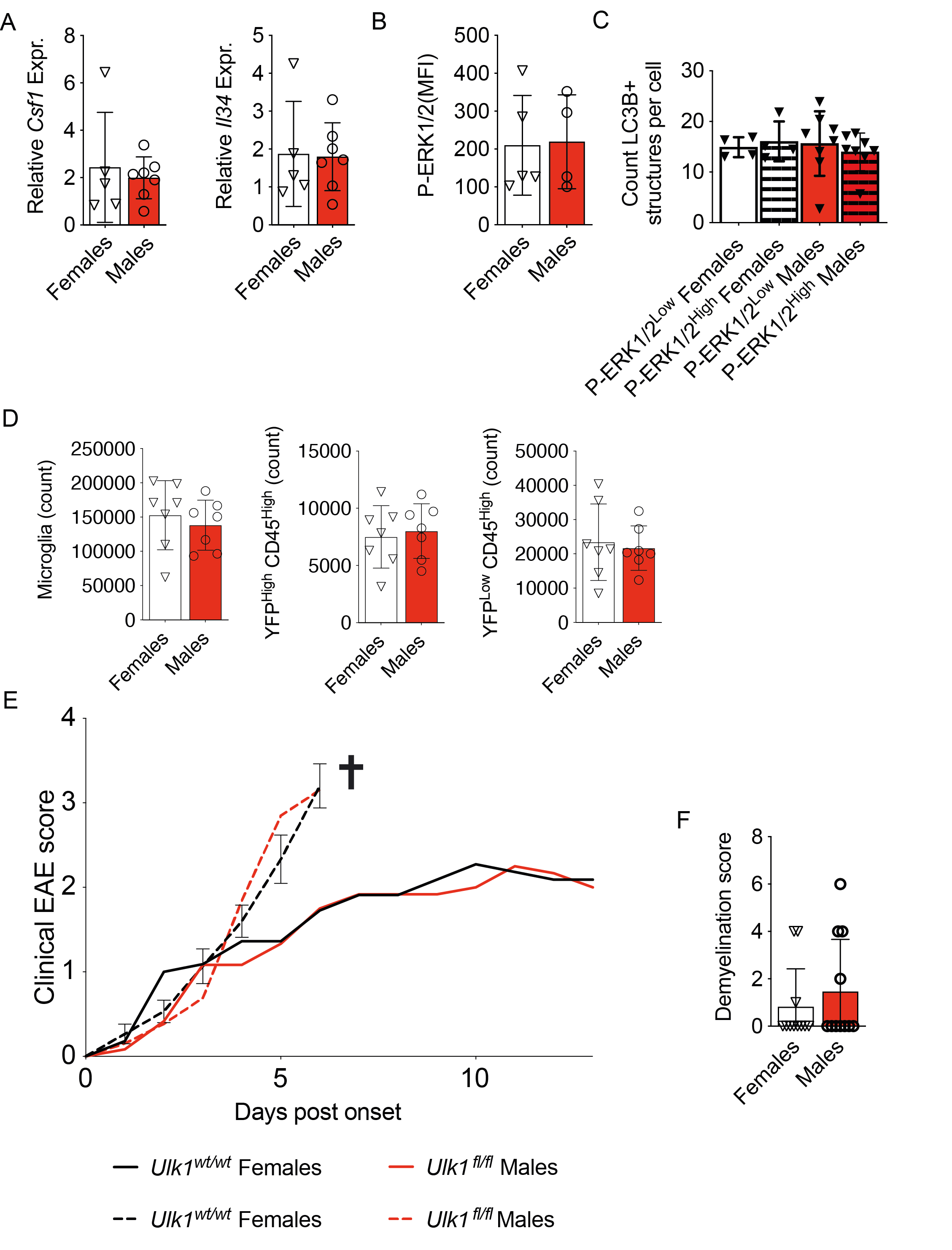


Supplementary figure 4

(A) CNS mRNA expression of *Csf1* and *Il34* normalized to *Hprt* and *B-actin* (females, *n*=5; males *n*=7). Rearranged data from figure 1D.

(B) Microglia phosphorylation status assessed ex vivo after 4h culture in CSF-1 enriched medium (females, *n* =5; males, *n* =4). Rearranged data from figure 1G.

(C) Quantification of LC3B+ autophagosomal structures in mi­­­­­­­­­croglia defined by the P-ERK1/2 level (females, *n* =4; males, *n* =8). Rearranged data from figure 3D.

(D) Flow cytometry quantification of CNS CD45^+^ populations where CD45^Intermediate^ YFP^+^ represent microglia targeted by the Tamoxifen induced *Ulk1* deletion. The YFP+ CD45^High^ populations are considered bone marrow derived myeloid cells and YFP- CD45+ other immune cells (all conditions, *n* =7)*.* Rearranged data from figure 4F.

(E) Clinical scores (left) and a graphic summary of aged mice with MOG-induced EAE (*Ulk1^wt/wt^* females *n* =11, *Ulk1^wt/wt^* males *n* =12; *Ulk1^fl/^*^fl^ females *n* =15; *Ulk1^fl/^*^fl^ males *n* =13). Rearranged data from figure 7A.

(F) Immunohistochemical images of and analysis of brain and spinal cord sampled day 15 post EAE induction. Black arrowheads indicate inflammatory infiltrates (females, *n* =11; males, *n* =11). Rearranged data from figure 6D.

(A) - (B), (D) and (E) - (F) Mann-Whitney Two-tailed *U*-test. Error bars represent mean + SD. (C) One-way Anova with Tukey posttest. Error bars represent mean + SD. Source data are provided as a Source Data file.
