# Supplementary material for: The aging mouse CNS is protected by an autophagy-dependent microglia population promoted by IL-34
Source: Nat Commun. 2024 Jan 9;15:383. doi: 10.1038/s41467-023-44556-6 (PMC10776874; doi:10.1038/s41467-023-44556-6)
Supplement: Supplementary file 3 — Description of Additional Supplementary Files [file 41467_2023_44556_MOESM3_ESM.docx]

**Description of Additional Supplementary Files**

Supplementary data 1: Raw and normalized Nanostring^TM^  nCounter Neoroinflammation panel RNA expression data (adult *n*=4; aged P-ERK1/2^Low^, *n*=4; aged P-ERK1/2^High^, *n*=4).

Supplementary data 2: Technical data.
